# Possible Therapeutic Strategy Involving the Purine Synthesis Pathway Regulated by ITK in Tongue Squamous Cell Carcinoma

**DOI:** 10.3390/cancers13133333

**Published:** 2021-07-02

**Authors:** Kaoru Onidani, Nami Miura, Yuki Sugiura, Yuichi Abe, Yukio Watabe, Takanori Kakuya, Taisuke Mori, Seiichi Yoshimoto, Jun Adachi, Takao Kiyoi, Yasuaki Kabe, Makoto Suematsu, Takeshi Tomonaga, Takahiko Shibahara, Kazufumi Honda

**Affiliations:** 1 Department of Biomarkers for Early Detection of Cancer, National Cancer Center Research Institute, Tokyo 104-0045, Japan; onidanikaoru@tdc.ac.jp (K.O.); namiura@ncc.go.jp (N.M.); yukio_watabe@tmhp.jp (Y.W.); takakuya18@gmail.com (T.K.); 2 Department of Oral and Maxillofacial Surgery, Tokyo Dental College, Tokyo 101-0061, Japan; sibahara@tdc.ac.jp; 3 Department of Biochemistry, Keio University School of Medicine, Tokyo 160-8582, Japan; yuki.sgi@keio.jp (Y.S.); ykabe@keio.jp (Y.K.); gasbiology@keio.jp (M.S.); 4 Laboratory of Proteome Research, National Institute of Biomedical Innovation, Health and Nutrition, Ibaraki, Osaka 567-0085, Japan; y.abe@aichi-cc.jp (Y.A.); jun_adachi@nibiohn.go.jp (J.A.); tomonaga@nibiohn.go.jp (T.T.); 5 Division of Pathology and Clinical Laboratory, National Cancer Center Hospital, Tokyo 104-0045, Japan; tamori@ncc.go.jp; 6 Department of Head and Neck Surgery, National Cancer Center Hospital, Tokyo 104-0045, Japan; seyoshim@ncc.go.jp; 7 Research and Development, Carna Biosciences, Inc., Kobe 650-0047, Japan; takao.kiyoi@carnabio.com; 8 Japan Agency for Medical Research and Development: AMED-CREST, AMED, Tokyo 104-0004, Japan; 9 Department of Bioregulation, Graduate School of Medicine, Nippon Medical School, Tokyo 113-8602, Japan

**Keywords:** tongue squamous cell carcinoma, IL2-inducible T-cell kinase (ITK), trifunctional purine biosynthetic protein adenosine-3 (GART)

## Abstract

**Simple Summary:**

We identified overexpression of interleukin-2–inducible T-cell kinase (ITK) as a novel biomarker for predicting the prognosis of tongue squamous cell carcinoma patients with poor outcomes. Oral cancer cell lines overexpressing ITK exhibited significantly increased proliferation in three-dimensional culture assays and murine inoculation models as compared with mock control cells. Phosphorylation proteomics analysis revealed that ITK expression induces the phosphorylation of a novel tyrosine residue of the trifunctional purine biosynthetic protein adenosine-3 (GART), an enzyme in the purine biosynthesis pathway. A significant increase in de novo purine biosynthesis was observed in cells expressing ITK. These results suggest that ITK is involved in enhancing the proliferation of cancer cells in the malignant phenotype by activating de novo purine biosynthesis through phosphorylation of GART. In this study, we report a possible therapeutic strategy involving the purine synthesis pathway regulated by ITK in tongue squamous cell carcinoma.

**Abstract:**

The epidermal growth factor receptor is the only available tyrosine kinase molecular target for treating oral cancer. To improve the prognosis of tongue squamous cell carcinoma (TSCC) patients, a novel molecular target for tyrosine kinases is thus needed. We examined the expression of interleukin-2–inducible T-cell kinase (ITK) using immunohistochemistry, and the biological function of ITK was investigated using biochemical, phosphoproteomic, and metabolomic analyses. We found that ITK is overexpressed in TSCC patients with poor outcomes. The proliferation of oral cancer cell lines expressing ITK via transfection exhibited significant increases in three-dimensional culture assays and murine inoculation models with athymic male nude mice as compared with mock control cells. Suppressing the kinase activity using chemical inhibitors significantly reduced the increase in cell growth induced by ITK expression. Phosphoproteomic analyses revealed that ITK expression triggered phosphorylation of a novel tyrosine residue in trifunctional purine biosynthetic protein adenosine-3, an enzyme in the purine biosynthesis pathway. A significant increase in de novo biosynthesis of purines was observed in cells expressing ITK, which was abolished by the ITK inhibitor. ITK thus represents a potentially useful target for treating TSCC through modulation of purine biosynthesis.

## 1. Introduction

A number of molecular-targeted drugs, including kinase-targeted drugs, are used clinically in the treatment of many types of cancer to improve the survival time of patients. However, cetuximab, a monoclonal antibody that blockades the epidermal growth factor receptor (EGFR), is the only kinase-targeted drug available for treating tongue squamous cell carcinoma (TSCC) [1]. Thus, additional therapeutic strategies focused on the inhibition of a wide variety of kinases are needed to treat patients more effectively with TSCC.

Interleukin-2–inducible T-cell kinase (ITK) is a member of the Tec family of non-receptor tyrosine kinases and is expressed in T-cells and mast cells. In T-cells, ITK functions downstream of the T-cell receptor and plays an important role in T-cell activation, development, differentiation, and production of pro-inflammatory cytokines [2]. It was recently reported that ITK gene expression is higher in tumor tissues than in normal tissues and associated with poor prognosis in head and neck cancer [3]. ITK protein expression reportedly increases with nevus to metastatic melanoma progression and is associated with tumor development and progression in melanoma [4]. However, little is known about ITK protein expression and function in almost all types of solid tumors. Trifunctional purine biosynthetic protein adenosine-3 (GART) is an enzyme in the de novo purine biosynthesis pathway. Purine metabolism is reportedly enhanced in proliferating cancer cells [5]. However, the mechanism of the de novo purine biosynthesis pathway through GART in TSCC has not been elucidated in detail.

In our previous study, we identified a useful prognostic biomarker for TSCC [6] and investigated the expression profiles of potential molecular drug targets in the treatment of some types of cancer [7,8]. In this study, we examined whether ITK expression is associated with prognosis of TSCC patients with the aim of identifying therapeutic molecular targets for TSCC. In addition, phosphoproteomic and metabolomic analyses were performed to investigate the mechanism of cancer cell proliferation via ITK and the de novo purine biosynthesis pathway via GART phosphorylation.

## 2. Materials and Methods

### 2.1. Patients and Tissue Samples

We examined a total of 86 patients who underwent glossectomy alone with curative intention for stage-I/-II TSCC at the National Cancer Center (NCC) Hospital (Tokyo, Japan) and Tokyo Dental College (TDC) (Tokyo, Japan). Glossectomy was categorized as class I or class II [9]. The 86 patients underwent glossectomy between 1999 and 2011, and the follow-up time for the 86 cases ranged from 7.2 to 188.4 months between 2000 and 2017, with a median follow-up time of 65.98 months. The study was performed with the approval of the Internal Review Board of the NCC and TDC regarding ethical issues (approval numbers: NCC, 2010-077; TDC, 446). This study was carried out in accordance with the Declaration of Helsinki.

### 2.2. Immunohistochemistry (IHC)

Formalin-fixed, paraffin-embedded TSCC tissues and animal experiment samples were cut into 4 μm sections. IHC was performed using an anti-ITK rabbit monoclonal antibody (clone Y401, Abcam, Cambridge, UK). ITK was immunostained using a Ventana DABMap detection kit and automated slide stainer (Discover XT; Ventana Medical System, Roche Diagnostics, Tucson, AZ, USA) [10,11]. ITK immunoreactivity was classified as either positive or negative. The ITK-positive group was defined as cases in which the staining area where the anti-ITK rabbit monoclonal antibody had been applied reacted stronger or equally with ITK in comparison with lymphocytes on the same slide glasses and encompassed >30% of the tumor area. In contrast, the ITK-negative group was defined as cases in which the ITK staining area encompassed <30% of the tumor area and demonstrated less reactivity compared with the lymphocytes [10,12]. Samples in which the ITK staining area encompassed <30% of the tumor area but exhibited stronger or equal reactivity compared with the lymphocytes were classified as negative. The positive control for the anti-ITK rabbit monoclonal antibody was lymphocytes. Staining patterns were evaluated by three independent investigators (Y.W., T.K., and K.O.) who had no clinical information regarding the cases.

### 2.3. Cell Lines, Antibodies, and Drugs

SAS, OSC-19, SCC-4, HSC-2, HSC-3, HSC-4, and KOSC-2 cl3-43 (KOSC2) human oral cancer cells and human embryonic kidney 293 (HEK 293) cells were purchased from the Japanese Collection of Research Bioresources Cell Bank (Osaka, Japan). The Jurkat E6.1 (Jurkat) human T-cell lymphoma cell line was purchased from KAC Co., Ltd. (Kyoto, Japan). SAS, KOSC2, and Jurkat cells were grown in RPMI supplemented with 10% FBS (Thermo Fisher Scientific, Waltham, MA, USA). Anti-GART rabbit monoclonal antibody (H8132) for western blot analysis and anti–β-actin mouse monoclonal antibody (ab6276) were purchased from Abcam (Cambridge, UK). Anti-GART mouse monoclonal antibody (4D6-1D5) (Abnova, Taipei, Taiwan) for immunofluorescence cytochemistry, anti-BTK mouse monoclonal antibody (ab54319), and anti-Bmx goat polyclonal (sc-8874) (Santa Cruz Biotechnology, Dallas, TX, USA) antibodies were purchased from the suppliers indicated. ITK inhibitor (Cmpd-5) was obtained from Carna Biosciences, Inc. (Hyogo, Japan) (Figure S1).

### 2.4. Establishment of Stably Infected Cells

Lentiviral particles for human ITK (LPP-A0222-Lv213-050) and the control (LPP-MCHR-Lv105-100-C) in the pEZ-Lv213 vector were purchased from GeneCopoeia (Rockville, MD, USA). SAS and KOSC2 cells were infected with lentiviral particles, and 24 to 48 hours after infection, the cells were selected with 1 μg/μL puromycin (Sigma-Aldrich, St. Louis, MO, USA). After 2 weeks of selection in puromycin, ITK expression was confirmed by western blot analysis. The cells were cultured in medium containing 1 μg/μL puromycin.

### 2.5. Western Blot Analysis

Proteins were extracted using Mammalian Protein Extraction Reagent (Thermo Fisher Scientific) containing a protease inhibitor mixture (Wako Pure Chemical Industries, Ltd., Osaka, Japan) at a ratio of 1:100. Protein samples were fractionated by sodium dodecyl sulfate–polyacrylamide gel electrophoresis and blotted onto polyvinylidene difluoride membranes (Merck Millipore, Darmstadt, Germany). Hybridization of antibodies and immunoblotting detection followed previously described methods [13].

### 2.6. In Vitro Cell Growth Assay

Cells stably expressing ITK and mock control cells (2 × 10^3^ cells per well in a 96-well plate) were seeded onto a NanoCulture Plate (Scivax, Boston, MA, USA) for 3D cell culture and cultured for an additional 3 or 4 days. Cell viability was evaluated using a RealTime-Glo MT Cell Viability Assay (Promega, Madison, WI, USA) [13,14].

### 2.7. Animal Experiments

Athymic male nude mice (BALB/c-nu; aged 5 weeks) were purchased from Charles River Laboratories Japan (Kanagawa, Japan) and maintained in a specific pathogen-free environment. The temperature in the animal facilities was 22 ± 0.5 °C, and the humidity was 45–65%. Light cycle was 12 hours. The cage was made of plastic, and the bedding material was wood chips. Food and water were freely available, and health status was monitored daily. The average body weight of 42 mice was 23.3 g (range, 20.4–26.5 g). Forty-two mice were randomly divided into two groups. SAS ITK-expressing and mock control cells (1 × 10^6^) were injected subcutaneously. Five days after subcutaneous injection, mice within 1 standard deviation of the average tumor volume of each group were selected (ITK-expressing cells, 19 mice; mock control cells, 14 mice), and tumor volume measurement was started. Tumors were measured daily using calipers, and tumor volume was calculated as follows: (width^2^) × (length)/2. Eight days after the start of tumor volume measurement, the mice were sacrificed when the tumors showed signs of ulcerations. No deaths were observed in the two groups. Animals were euthanized by isoflurane anesthesia followed by cervical dislocation. Tumor samples were removed, formalin fixed, paraffin embedded, and cut into sections. Sections were immunohistochemically stained with an anti-ITK rabbit monoclonal antibody. All animal experimental procedures were reviewed and approved by the ethics committee of the National Cancer Center Research Institute (Tokyo, Japan).

### 2.8. Preparation and Digestion of Protein Lysate

The cells were harvested after washing with ice-cold PBS containing PhosSTOP phosphatase inhibitor cocktail and protease inhibitor cocktail, complete EDTA-free (Roche Diagnostics). Collected cells were pelleted using centrifugation and quickly frozen in liquid nitrogen. Cell pellets were stored at −80°C until LC-MS/MS analysis.

Frozen pellets were solubilized using phase transition surfactant buffer (50 mM ammonium bicarbonate, 12 mM sodium deoxycholate, 12 mM sodium lauryl sarcosinate) supplemented with PhosSTOP and complete protease inhibitors. The protein concentration of the lysate was determined using a detergent-compatible protein assay (Bio-Rad, Berkeley, CA, USA). A total of 2 mg of the protein was used for subsequent analyses. Reduction, alkylation, and digestion of the sample were conducted as previously described [15]. Removal of surfactant in the samples was carried out as previously reported [15]. The samples were subjected to an additional desalting step using an OASIS HLB column (Waters Corp., Milford, MA, USA).

Primary enrichment of phosphopeptides using an Fe-IMAC column and TMT labeling and enrichment of phosphotyrosine peptides by immunoprecipitation are described previously [8].

### 2.9. Mass Spectrometry Analysis for Quantitative Phosphotyrosine Proteomics

Phosphotyrosine proteomics was performed on a Q-Exactive Plus mass spectrometer (Thermo Fisher Scientific) coupled to an Ultimate 3000 liquid chromatograph (Thermo Fisher Scientific). The observed condition of peptide separation on the analytical column corresponded to previous results [15]. The mass spectrometry parameters were the same as those described in the previous study [15]. LC-MS/MS analysis of these samples was performed at the National Institute of Biomedical Innovation, Health and Nutrition (Osaka, Japan).

### 2.10. Identification and Quantification of Phosphopeptides

Raw files from the Q-Exactive Plus were subjected to peptide identification with Maxquant, version 1.5.1.2 (Max Planck Institute, Munich, Germany) [16]. Searching of MS/MS spectra against the UniProt human database (release 2017/01) combined with 262 common contaminants was performed. For quantification with TMT reagents, TMT tags on lysine residues and peptide N-termini were set as fixed modifications. The false discovery rate in peptides was set to <1% using a decoy search against a reverse database. The other peptide identification parameters were consistent with the previous report [15]. Quantitative data from each phosphosite were log2 transformed and normalized using median centering of the values in each TMT label.

### 2.11. Bioluminescence Resonance Energy Transfer (BRET) Assay

Open reading frame clones of ITK (pFN21AB6113) and GART (pFN21AB3014) were purchased from Kazusa DNA Research Institute (Chiba, Japan). NanoLuc (pFN31K)-GART, NanoLuc (pFN31K)-ITK, HaloTag (pFN21A)-GART, and HaloTag (pFN21A)-ITK fusion vectors were constructed using the Flexi Vector system (Promega). HaloTag control vector was purchased from Promega. HEK 293 cells were transfected with a DNA mixture consisting of the ITK or GART fusion vector and HaloTag control vector using Fugene HD (Promega). BRET detection followed using a NanoBRET Nano-Glo Detection system and a GloMax Discover System according to the manufacturer’s (Promega) instructions. BRET ratios are expressed as milliBRET units (mBU), in which 1 mBU corresponds to the corrected BRET ratio × 1000 [17].

### 2.12. Immunofluorescence Cytochemistry

HEK 293 cells transfected with a DNA mixture consisting of ITK fusion vector or HaloTag control vector were seeded on poly-L-lysine-coated cover glasses (BD Biosciences, San Jose, CA, USA) and fixed with 4% paraformaldehyde for 15 minutes at room temperature. The cells were incubated with anti-ITK rabbit monoclonal antibody and anti-GART mouse monoclonal antibody at 4 °C overnight. After incubation with Alexa Fluor 488–conjugated goat anti-rabbit IgG (A21434, Thermo Fisher Scientific) and Alexa Fluor 594–conjugated goat anti-mouse IgG (A11032, Thermo Fisher Scientific), the specimens were observed under a confocal laser scanning microscope (SLM 880 with Airyscan, ZEISS, Bio-Rad Laboratories, Tokyo, Japan) [13,18,19].

TSCC and animal experiment sections were deparaffinized, and the target antigens were activated. Anti-CD4 antibody (MA5-12259, Thermo Fisher Scientific), anti-Ki67 antibody (14-5698-82, Thermo Fisher Scientific), and anti-ITK rabbit monoclonal antibody (clone Y401, ab32039, Abcam) were used in dilutions, followed by reaction with Alexa Fluor 594–conjugated goat anti-mouse IgG (A11032, Thermo Fisher Scientific), Alexa Fluor 555–conjugated goat anti-rat IgG (A21434, Thermo Fisher Scientific), and Alexa Fluor 488–conjugated goat anti-rabbit IgG (A21434, Thermo Fisher Scientific). Finally, sections were mounted with DAPI using Vectashield (H-1200, Vector Laboratories, Inc., Burlingame, CA, USA). Images were acquired using a Virtual Slide Scanner (NanoZoomer 2.0-HT; Hamamatsu Photonics) [7] and fluorescence microscope (ImagerZ; Carl Zeiss AG, Oberkochen, Germany).

### 2.13. Metabolomic Analysis

A total of 4 × 10^6^ SAS cells per dish were seeded in 10-cm culture dishes and incubated overnight. In experiments using the ITK inhibitor Cmpd-5, after overnight incubation, cells were treated with 0.8 µM Cmpd-5 (Carna Biosciences) and incubated for 24 hours. For metabolic pathway tracing experiments, the culture medium was exchanged for a glucose-free RPMI containing 4 g/L [U-^13^C]-glucose (Sigma-Aldrich, Castle Hill, Australia). Cells were washed twice with PBS, and after thorough removal of the washing buffer, the dishes were immediately frozen in liquid nitrogen.

Metabolite extraction from cultured cells was performed as described previously [20]. Briefly, frozen cells were lysed and scraped from the dish using ice-cold methanol (500 μL) together with internal standard compounds (see below), followed by the addition of an equal volume of chloroform and 0.4 times the volume of ultrapure water (LC/MS grade, Wako). The suspension was then centrifuged at 15,000 rpm for 15 min at 4 °C. After centrifugation, the aqueous phase was ultrafiltered using an ultrafiltration tube (Ultrafree MC-PLHCC, Human Metabolome Technologies, Yamagata, Japan). The filtrate was concentrated using a vacuum concentrator (SpeedVac, Thermo Fisher Scientific), and the concentrated filtrate was dissolved in 50 μL of ultrapure water and used for mass spectrometry analyses.

We used 2-morpholinoethanesulfonic acid as an internal standard compound (added to methanol for metabolite extraction) for anionic metabolites. These compounds are not present in tissues; thus, they serve as ideal standards. Loss of endogenous metabolites during sample preparation was corrected by calculating the recovery rate (%) for each sample measurement.

### 2.14. Ion Chromatography-Tandem Mass Spectrometry for Anionic Metabolites

For metabolomic analyses focused on glucose metabolism central pathways, namely glycolysis, TCA-cycle, and pentose phosphate pathway, anionic metabolites were measured using an Orbitrap-type mass spectrometer (Q-Exactive focus, Thermo Fisher Scientific) connected to a high-performance ion chromatography (IC) system (ICS-5000+, Thermo Fisher Scientific) that enabled us to perform highly selective and sensitive metabolite quantification based on the IC-separation and Fourier transform MS principle [21].

The IC was equipped with an anion electrolytic suppressor (Dionex AERS 500, Thermo Fisher Scientific) to convert the potassium hydroxide gradient into pure water before the sample entered the mass spectrometer. The separation was performed using a Thermo Fisher Scientific Dionex IonPac AS11-HC, 4-μm particle-size column. The IC flow rate was 0.25 mL/min supplemented post-column with a 0.18 mL/min makeup flow of MeOH. The potassium hydroxide gradient conditions for IC separation were as follows: from 1 mM to 100 mM (0–40 min), 100 mM (40–50 min), and 1 mM (50.1–60 min), at a column temperature of 30 °C.

The Q Exactive focus mass spectrometer was operated under ESI negative mode for all detections. Full mass scan (*m/z* 70−900) was used at a resolution of 70,000. The automatic gain control target was set at 3 × 10^6^ ions, and maximum ion injection time was 100 ms. Source ionization parameters were optimized with the spray voltage at 3 kV and other parameters as follows: transfer temperature at 320 °C, S-lens level at 50, heater temperature at 300 °C, sheath gas at 36, and Aux gas at 10.

### 2.15. Growth Inhibition Analysis with ITK Inhibitor

Cells stably expressing ITK and mock control cells (2 × 10^3^ cells or 5 × 10^3^ cells per well in a 96-well plate) were seeded in a NanoCulture plate for 3D cell culture and incubated for 6 hours. ITK inhibitor was adjusted to 1.8 μM. Next, half of the medium was aspirated, and the adjusted ITK inhibitor was added until the final concentration of ITK inhibitor was 0.8 μM. After 144 hours, cell viability was evaluated using a RealTime-Glo™ MT Cell Viability Assay (Promega).

### 2.16. Statistical Analysis

The significance of differences was assessed using the Student’s *t*-test, Welch’s *t*-test, Pearson’s χ^2^ test, and Fisher’s exact test. Overall survival (OS) was measured as the period from surgery to the date of death or last follow-up. Disease-free survival (DFS) was defined as the period from surgery to the date of relapse, second primary cancer, death from any cause, or last follow-up. Survival rates were estimated by the Kaplan–Meier method. Differences between OS or DFS curves were assessed using the log-rank test. Univariate and multivariate analyses were performed using the Cox regression model. The multivariate model was adjusted for age, stage, and ITK expression. Data were analyzed using StatFlex statistical software package, version 6.0 (StatFlex, Osaka, Japan), and SAS software, version 9.4 (SAS Institute, Cary, NC, USA). A *p*-value of < 0.05 was considered statistically significant.

## 3. Results

### 3.1. ITK Protein Expression in Patients with TSCC

**Figure 1:**
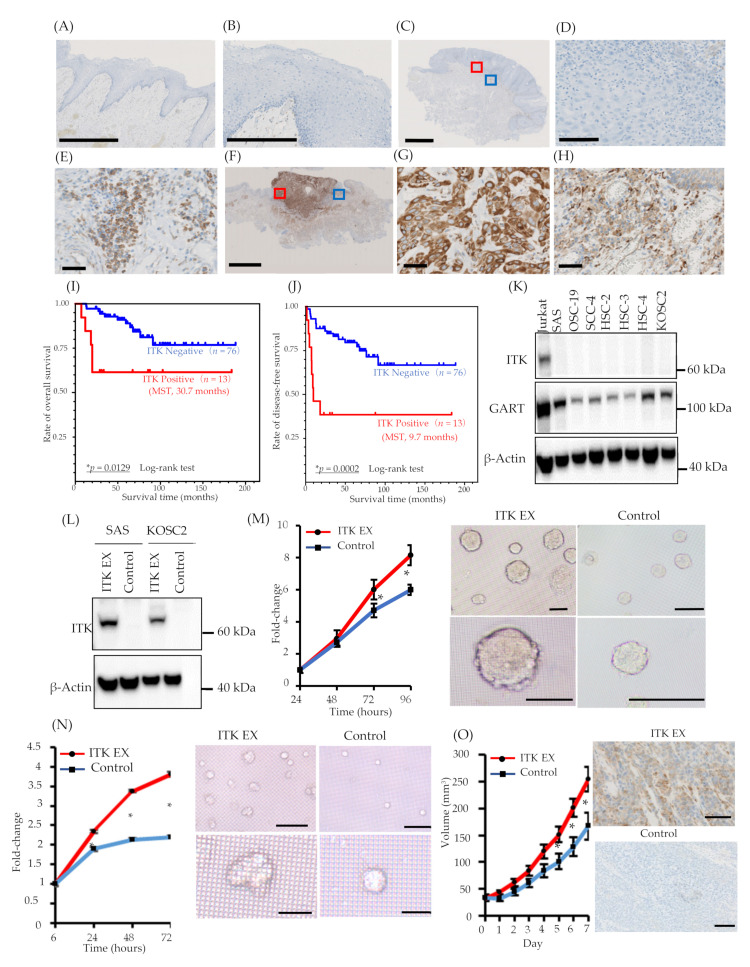
Expression of interleukin-2–inducible T-cell kinase (ITK) in surgically removed tongue squamous cell carcinoma tissue, correlation between ITK expression and prognosis, and proliferation of oral cancer cell lines associated with ITK expression in vitro and in vivo. (**A** and **B**) Representative expression of ITK protein in the normal tongue mucosa (bar, 1 mm) (**A**). (**B**) shows a magnified image of the normal tongue mucosa (bar, 250 μm). (**C–E**) Representative images of ITK-negative cases. (B: bar, 2.5 μm; C: bar, 100 μm; D: bar, 50 μm). Red square denotes the part of the cancer lesion image that was magnified (**D**). Blue square denotes the part of the lymphocyte image that was magnified (E). (**F–H**) Representative images of ITK-positive cases (E: bar, 5 μm; F and G: bar, 50 μm). Red square denotes the part of the cancer lesion image that was magnified (**G**). Blue square denotes the part of the lymphocyte image that was magnified (**H**). (**I**) OS curves for the ITK-positive (red line, *n* = 13) and ITK-negative (blue line, *n* = 76) subgroups (*p* = 0.0129). (**J**) DFS curves for the ITK-positive (red line, *n* = 13) and ITK-negative (blue line, *n* = 76) subgroups (*P* = 0.0002). Statistical significance was assessed using the log-rank test. (**K**) Western blot analysis of ITK and GART expression in oral cancer cells. β-Actin was blotted as a loading mock control. (**L**) Western blot analysis of ITK expression in two lines of oral cancer cells that stably express ITK (ITK EX) and mock control cells (Control). β-Actin was blotted as a loading control. (**M**) Proliferation of ITK-expressing SAS cells and mock control cells and representative photographs of ITK-expressing SAS cell spheroids and mock control cell spheroids after 96 hours of culture. Top panels are overall views (bars, 200 μm); bottom panels are magnified images (bars, 200 μm). (**N**) Proliferation of ITK-expressing KOSC2 cells and mock control cells and representative photographs of ITK-expressing KOSC2 cell spheroids and mock control cell spheroids after 72 hours of culture. Top panels are overall views (bars, 200 μm); bottom panels are magnified images (bars, 50 μm). (**O**) Tumor volume of ITK-expressing SAS cells and mock control cells in an inoculation model involving subcutaneous injection of SAS cells in nude mice, and immunohistochemistry using an anti-ITK antibody of transplanted ITK-expressing cells and mock control cells in mice (bars, 100 μm). Error bars indicate standard error. * *p* < 0.01 (Student’s *t*-test).

The expression of ITK protein in patients with TSCC was examined using IHC with an anti-ITK rabbit monoclonal antibody that was purchased from CST (details described in the ‘Methods’ section; antibody clone number Y401). First, we confirmed the specificity of the anti-ITK rabbit monoclonal antibody and observed no cross-reactivity for other molecules of the Tec family, as illustrated in Figure S2. Expression of ITK protein was not observed in normal tongue mucosa adjacent to the tumor (Figure 1A,B), and lymphocytes were stained by the ITK antibody in all 86 patient TSCC tissues on the same pathology sections as the internal controls (Figure 1E,H). Immunofluorescence double staining was performed with anti-CD4 antibody and anti-ITK rabbit monoclonal antibody (Figure S3). ITK-positive CD4^+^ T-cells were stained with both anti-CD4 antibody and anti-ITK rabbit monoclonal antibody. Of 86 patients with TSCC, 73 (84.9%) were ITK negative (Figure 1C,D), and 13 (15.1%) were ITK positive (Figure 1F,G). The Kaplan–Meier curves showed a statistically significant difference between the ITK-negative and ITK-positive groups in both OS (log-rank trend test *P* = 0.0129) (Figure 1I) and DFS (log-rank trend test *P* = 0.0002) (Figure 1J). The correlations between clinical findings and ITK expression pattern are summarized in Table 1. Statistically significant differences were observed between ITK expression and mode of invasion (*P* = 0.0000), late metastasis of cervical lymph nodes (*P* = 0.0006), and lymphovascular invasion (*P* = 0.0101). No statistically significant associations were observed between ITK expression and age, sex, clinical stage, clinical histologic differentiation (poorly/moderately differentiated versus well-differentiated), or perineural invasion (Table 1).

### 3.2. Hazard Ratios (HRs) for Death and Prognostic Significance of ITK Protein Expression in TSCC

We calculated the HR for death with respect to various parameters, including age, clinical stage, ITK protein expression, sex, histologic differentiation, mode of invasion, late metastasis of cervical lymph nodes, lymphovascular invasion, and perineural invasion, using univariate and multivariate Cox regression analyses. Univariate analyses revealed that ITK protein expression, histologic differentiation, mode of invasion, late metastasis of cervical lymph nodes, and lymphovascular invasion exhibited statistically significant HRs for death: 3.544 (95% confidence interval [CI] 1.225–10.247) for ITK protein expression, 3.401 (95% CI 1.264–9.149) for histologic differentiation, 5.112 (95% CI 1.768–14.783) for mode of invasion, and 4.497 (95% CI 1.684–12.010) for lymphovascular invasion (Table 2). Multivariate analyses indicated that ITK protein expression was an independent positive predictor of death in TSCC patients: HR 3.167 (95% CI 1.089–9.215) (Table 2).

### 3.3. Role of ITK in Cancer Cell Proliferation In Vitro and In Vivo

ITK expression was not detected in the oral cancer cell lines we investigated (Figure 1K). Consequently, we engineered SAS and KOSC2 oral cancer cells that stably express ITK (Figure 1L) and investigated the effect of ITK expression on cell proliferation in three-dimensional (3D) cultures. Proliferation of ITK-expressing SAS (Figure 1M) and KOSC2 (Figure 1N) cells was significantly enhanced compared with mock control cells. Figure panels 1M and 1N show photographs of spheroids of cultured SAS and KOSC2 cells, respectively. After 72 or 96 hours of culture, ITK-expressing cell spheroids were larger than mock control cell spheroids. We also investigated the effect of ITK expression on cell proliferation using in vivo models in which immunodeficient mice were inoculated subcutaneously with cancer cells. ITK-expressing SAS tumor cells grew faster than mock control cells (Figure 1O). Ki67 staining was stronger in ITK-expressing SAS tumor cells than in mock control cells (Figure S4). IHC confirmed the expression of ITK in tumor cells (Figure 1O). Neither ITK-expressing KOSC2 nor mock control cells colonized mice.

### 3.4. Proteomic Analysis of ITK Tyrosine Phosphorylation

**Figure 2:**
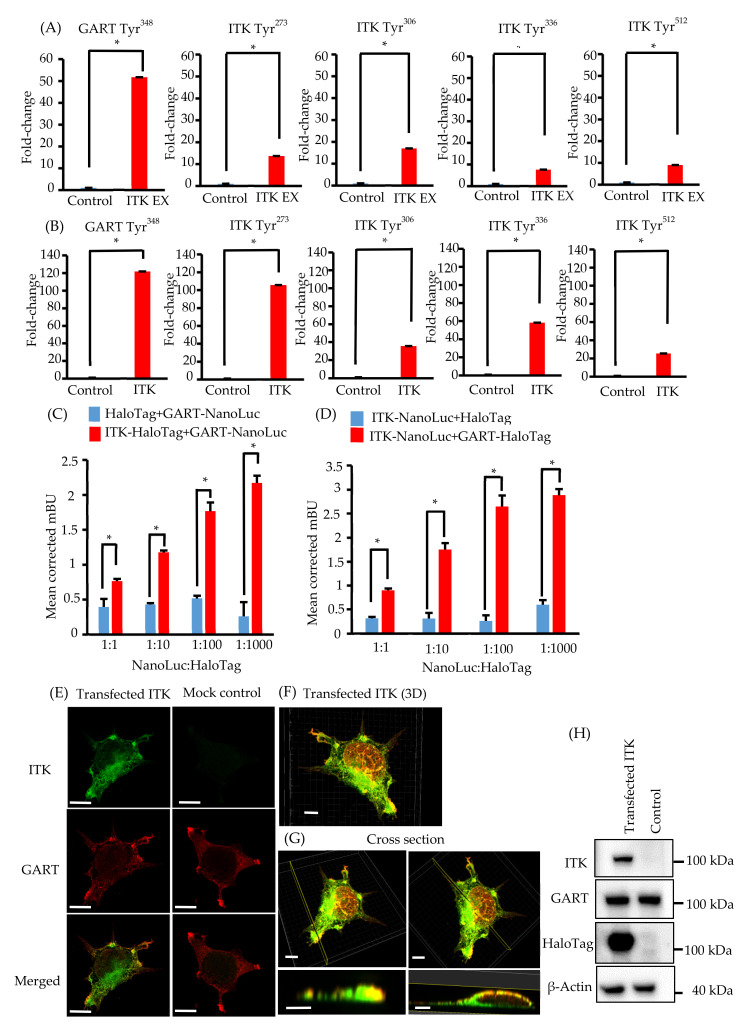
Phosphotyrosine proteomic analysis of interleukin-2–inducible T-cell kinase (ITK)-expressing cells and mock control cells, and protein–protein interaction between ITK and trifunctional purine biosynthetic protein adenosine-3 (GART). (**A,B**) Quantitative comparison of phosphotyrosine residues on GART and ITK in SAS cells (**A**) and KOSC2 cells (**B**). Error bars indicate standard deviation. * *p* < 0.05 (Student’s *t*-test). (**C**) BRET assay in HEK 293 cells. Red bars indicate HEK 293 cells transfected with HaloTag-fused ITK and NanoLuc-fused GART. Blue bars indicate HEK 293 cells transfected with HaloTag and NanoLuc-fused GART. (**D**) BRET assay in HEK 293 cells. Red bars indicate HEK 293 cells transfected with NanoLuc-fused ITK and HaloTag-fused GART. Blue bars indicate HEK 293 cells transfected with HaloTag and NanoLuc-fused ITK. Error bars indicate standard deviation. * *p* < 0.01 (Student’s *t*-test). (**E**) Immunofluorescence cytochemistry of HEK 293 cells with anti-ITK (green) and anti-GART (red) antibodies. ITK was transfected, and GART was the endogenous protein. Left panels show HaloTag-fused ITK-transfected HEK 293 cells. Right panels show HaloTag-transfected HEK 293 cells (bars, 5 μm). (**F** and **G**) 3D images of immunofluorescence cytochemistry with anti-ITK (green) and anti-GART (red) antibodies. These images show HaloTag-fused ITK-transfected HEK 293 cells. (F: bar, 5 μm; G: upper panel bars, 5 μm; lower panel bars, 3 μm). (**H**) Western blot analysis of ITK and GART expression in HaloTag-fused ITK-transfected or HaloTag-transfected HEK 293 cells as a mock control. β-Actin was blotted as a loading control.

Because ITK is a tyrosine kinase, we used mass spectrometry to investigate its tyrosine phosphorylation profile to identify novel biological behaviors in newly established oral cancer cell lines expressing ITK. Levels of tyrosine phosphorylation in SAS and KOSC2 cells were significantly higher in ITK-expressing cells than in mock control cells for 45 and 179 peptides, respectively (Tables S1 and S2). A total of 26 of the peptides were common to SAS and KOSC2 cells (Table S3). Excluding ITK peptides, GART peptides exhibited the highest levels of tyrosine phosphorylation among the 26 common peptides in ITK-expressing cells. GART and ITK phosphotyrosine residues common to SAS and KOSC2 cells are shown in Figure 2A,B. Compared with mock control cells, levels of a GART-associated specific phosphotyrosine (Tyr348) were significantly increased by 51- to 121-fold in ITK-expressing SAS and KOSC2 cells, respectively (Figure 2AB). Figure S5 shows tandem mass spectra representing the phosphorylated residues of GART.

### 3.5. Elucidation of the Association between ITK and GART

We investigated the direct association between ITK and GART, as GART is endogenously expressed in oral cancer cell lines and HEK 293 cells (Figure 1K and Figure 2H). In cells transfected with ITK or GART fused to NanoLuc or HaloTag, BRET occurred in a HaloTag concentration-dependent manner. By contrast, BRET was not detected in cells transfected with HaloTag not fused to ITK or GART (Figure 2C,D). Immunofluorescence analysis of the intracellular localization of ITK (green) and GART (red) (Figure 2E–G) indicated the partial colocalization of ITK and GART in HEK 293 cells (Figure 2E). Analyses of 3D images showed that ITK and GART interacted at the non-adherent surface of the cell or close to the tip of the cellar protrusions (Figure 2F,G). Expression of transfected ITK and endogenous GART in HEK 293 cells was confirmed by western blotting (Figure 2H).

### 3.6. Assessment of De Novo Synthesis of Purines in ITK-Expressing Cells

**Figure 3:**
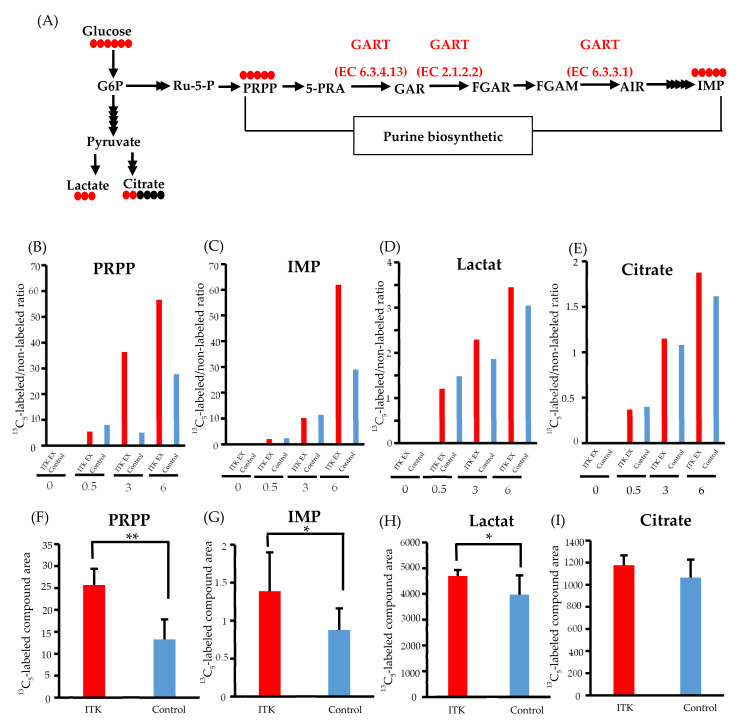
Metabolomic analysis of interleukin-2–inducible T-cell kinase (ITK)-expressing cells and mock control cells. (**A**) Purine biosynthesis pathway and glycolytic pathway (G6P, glucose 6-phosphate; Ru-5-P, ribulose-5-phosphate; PRPP, 5-phosphoribosyl 1-pyrophosphate; 5-PRA, 5-phosphoribosyl-1-amine; GAR, glycinamide ribonucleotide; FGAR and FGAM, formylglycinamide ribonucleotide; AIR, 5-aminoimidazole ribonucleotide; IMP, inosine monophosphate). Red and black circles indicate the number of ^13^C and ^12^C atoms in the metabolites, respectively. (**B–E**) Ratio of ^13^C- to ^12^C-labeled compounds at 0, 0.5, 3, and 6 hours after addition of ^13^C6-glucose in SAS cells (B: PRPP; C: IMP; D: lactate; E: citrate). (**F–I**) Absolute amount of each ^13^C-labeled compound at 0 and 6 hours after addition of ^13^C6 -glucose in SAS cells (F: PRPP; C: IMP; D: lactate; E: citrate). Error bars indicate standard deviation. * *p* < 0.05, ** *p* < 0.001 (Student’s *t*-test).

To clarify whether transfected ITK accelerates de novo synthesis of purines via activation of GART enzymatic activity, we performed ^13^C-glucose tracing analyses. After replacement of ^13^C6-glucose in the culture medium, we simultaneously quantified ^13^C-containing metabolites of the purine synthesis pathway, inosine monophosphate (IMP), and 5-phosphoribosyl-1-pyrophosphate (PRPP), as well as lactate and citrate (Figure 3A).

Time-course profiling of the ^13^C replacement rate (i.e., a ratio of ^13^C6-glucose to ^12^C6-glucose) indicated increased influx of ^13^C to the PRPP pool (>2-fold) between 3 and 6 hours post-replacement (Figure 3B). Such an increase in ^13^C influx into IMP was also demonstrated at 6 hours post-replacement, with an almost 2-fold increase (Figure 3C), while only small increases in the ^13^C replacement rate of lactate and citrate were observed (Figure 3D and E).

Increased purine de novo synthesis in ITK-expressing cells was confirmed by quantifying the amounts of ^13^C-labeled compounds (Figure 3F–I). Compared to mock control cells, the amounts of ^13^C5-PRPP, ^13^C5-IMP, and ^13^C3-lactate were significantly higher in ITK-expressing cells at 6 hours after replacement of ^13^C6-glucose (Figure 3F–H), while there was no statistically significant difference in citrate amount (Figure 3I).

### 3.7. Inhibition of CellG and Suppression of GART Phosphorylation and Purine Metabolism Using an ITK Inhibitor

**Figure 4:**
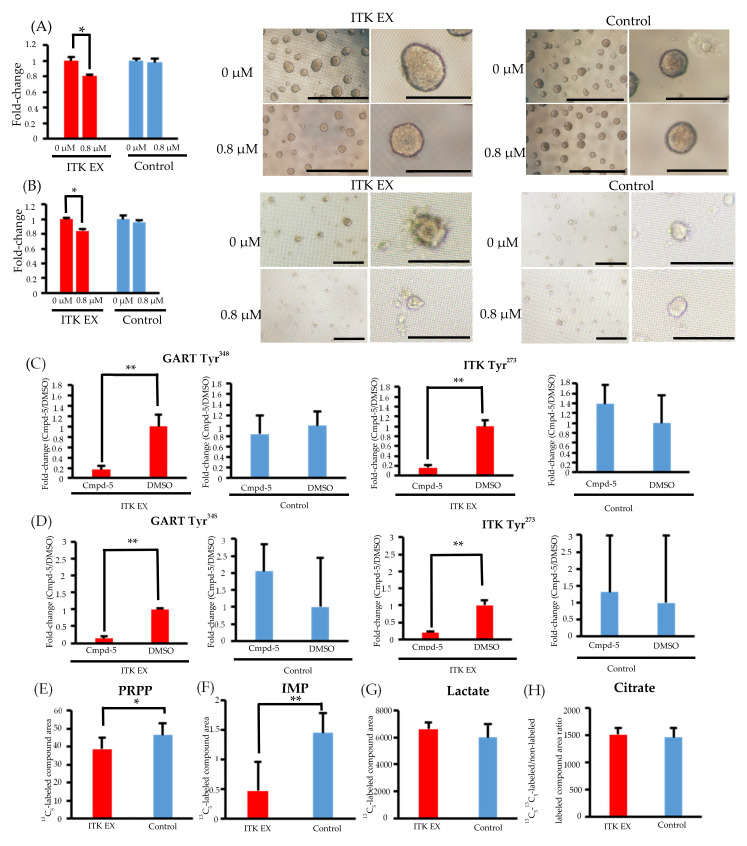
Effect of the interleukin-2–inducible T-cell kinase (ITK) inhibitor on ITK-expressing cells and mock control cells. (**A**) Rate of ITK-expressing SAS cells and mock control cells treated with DMSO or ITK inhibitor (Cmpd-5) for 144 hours. Images are representative photographs of ITK-expressing SAS cell spheroids and mock control cell spheroids treated with DMSO or ITK inhibitor for 144 hours. Left panels of ITK-expressing cell spheroids and mock control cell spheroids are overall views (bars, 1 mm), and right panels are magnified images (bars, 500 μm). * *p* < 0.001 (Student’s *t*-test) (**B**) Rate of ITK-expressing KOSC2 cells and mock control cells treated with DMSO or ITK inhibitor (Cmpd-5) for 144 hours. Images are representative photographs of ITK-expressing KOSC2 cell spheroids and mock control cell spheroids treated with DMSO or ITK inhibitor for 144 hours. Left panels of ITK-expressing cell spheroids and mock control cell spheroids are overall views (bars, 200 μm), and right panels are magnified images (bars, 50 μm). * *p* < 0.001 (Student’s *t*-test). (**C,D**) ITK-expressing cells and mock control cells were treated with DMSO or ITK inhibitor (Cmpd-5) for 24 hours, and phosphotyrosine residues on GART and ITK were examined (C: SAS cells; D: KOSC2 cells). ** *p* < 0.05 (Student’s *t*-test). (**E–H**) Absolute amount of each ^13^C-glucose metabolite at 0 and 6 hours after addition of ^13^C-glucose in ITK-expressing cells and mock control cells treated with ITK inhibitor (E: PRPP; F: IMP; G: lactate; H: citrate). * *p* < 0.01, ** *p* < 0.001 (Student’s *t*-test). Error bars indicate standard deviation.

In both SAS and KOSC2 cells, the ITK-associated increase in cell proliferation was diminished by treatment with the ITK inhibitor Cmpd-5 (Figure 4A,B). The chemical formula of Cmpd-5 is shown in Supplementary Figure S1. Spheroids of ITK-expressing cells treated with Cmpd-5 were smaller than those of untreated ITK-expressing cells. By contrast, the proliferation rate and spheroid volume of mock control cells were not affected by treatment with the ITK inhibitor (Figure 4A,B).

Phosphoproteomic analyses revealed that phosphorylation of GART and ITK was significantly suppressed in both ITK-expressing SAS and KOSC2 cells treated with Cmpd-5 compared with untreated cells, whereas the ITK inhibitor had no effect on GART and ITK phosphorylation in the mock control cells (Figure 4C,D and Figure S6). In metabolomic analyses with the ITK inhibitor, the absolute amounts of ^13^C5-PRPP and IMP were significantly lower in ITK-expressing cells treated with Cmpd-5 at 6 hours after addition of ^13^C6-glucose compared to mock control cells treated with the ITK inhibitor (Figure 4E,F). There were no statistically significant differences in the absolute amounts of ^13^C3- lactate and citrate (Figure 4G,H).

## 4. Discussion

More than 30 kinase-targeted drugs have received U.S. Food and Drug Administration approval for use in treating cancer, and additional kinase-specific inhibitors are in development [22]. However, only one kinase-targeted drug has been approved for use in treating oral cancers. In this study, we focused on ITK as a novel molecular target in TSCC, which is the most frequent oral cancer. We demonstrated that ITK expression is closely associated with poor clinical outcome in patients with stage I and II TSCC. Our data also showed that ITK enhances the proliferation of cultured cancer cells. Phosphoproteomic approaches are frequently used in research to discover therapeutic targets for molecular oncology. Our group has used phosphoproteomic approaches to explore molecular pathways to identify novel targets for inhibition using small compounds in cancer treatment. We found Src and related signaling pathways are activated in cetuximab-resistant colorectal cancer cell lines and therefore represent potential therapeutic targets [15].

ITK is a member of the Tec kinase family, and aberrant expression of ITK is involved in T-cell malignancies [23,24]. Previous studies examining ITK expression in solid tumors are limited to melanoma, in which aberrant expression was shown to be associated with poor outcome [4]. We therefore investigated the role of ITK in epithelial malignant tumors. Due to similarities in the amino acid sequences of Tec family kinases, we first confirmed the specificity of the anti-ITK antibody used in the study and found that it reacted specifically to ITK (Figure S1). This antibody is the same as that used in a study in which the correlations between ITK expression and melanoma were evaluated by IHC using clinical samples [4]. IHC using an anti-ITK antibody revealed aberrant expression of ITK protein in five patients with TSCC who had a significantly poor prognosis, a result similar to the previous melanoma study. We thus conclude that the results of IHC analyses of TSCC tissues using this anti-ITK antibody are reasonable and that the results of staining using the anti-ITK antibody are reliable.

To elucidate the biological role of ITK in the malignant phenotype of TSCC, we generated new lines of ITK-expressing oral cancer cells because we could not detect significant ITK expression in existing oral cancer cell lines. The results of BRET analyses revealed a direct interaction between ITK and GART. Our group has developed a high-throughput phosphoproteome analysis method to identify phosphopeptides at sufficient depth [25]. We previously used this technique to identify kinase targets for cancer treatment from cultured cells and trace amounts of biopsy samples [8,26]. In this study, significant increases (51- to 121-fold) in levels of tyrosine phosphorylation of GART were observed in ITK-expressing oral cancer cell lines. A phosphotyrosine proteomic analysis identified the phosphorylation site of GART as Tyr^348^. In previous mass spectrometry studies, Tyr^348^ in GART has been frequently identified in the PhosphoSitePlus database [27]. Phosphorylation of Tyr^348^ in GART was also reported in a human leukemia-derived T-cell line following stimulation with IL-2 or IL-15 [28]. These data also suggest that GART is a substrate for protein phosphorylation catalyzed by ITK.

GART is an enzyme in the de novo purine biosynthesis pathway [29]. In this pathway, six enzymes, including GART, catalyze the conversion of PRPP to IMP. Enzymes mediating de novo purine biosynthesis form dynamic multi-enzyme complexes, referred to as purinosomes, under conditions that stimulate purine biosynthesis [30]. GART is a core scaffolding protein of the purinosome, in conjunction with PRPP amidotransferase and phosphoribosyl formylglycinamidine synthase, which are the first enzymes in the de novo purine biosynthesis pathway [31]. Generation of IMP is promoted when purinosome formation increases [32]. Phosphorylation of CK2, a serine/threonine-selective protein kinase, has been associated with purinosome formation, and the core scaffolding proteins PRPP amidotransferase, GART, and formylglycinamidine synthase might serve as substrates for CK2 [29]. It was suggested that increases in PRPP and IMP in ITK-expressing oral cancer cells are due to the accelerated tyrosine phosphorylation of GART by ITK and activation of the de novo purine biosynthesis pathway. Indeed, it appears from the results of metabolomic analyses that the rates of change in the ^13^C_6_-glucose-labeled to non-labeled ratios in the purine metabolism pathway exhibited by PRPP and IMP in ITK-expressing cells were faster than those of the lactate and citrate pathways in comparison with mock control cells.

We conclusively demonstrated that compared to mock control cells, de novo purine biosynthesis was enhanced in the newly generated oral cancer cell lines that express ITK. De novo purine biosynthesis is reportedly enhanced in a variety of cancers due to demand for purine nucleotides for tumor cell proliferation [33], which suggests that the de novo purine biosynthesis pathway is a potentially important target in cancer treatment [34]. In experimental models using multiple cell lines, we found that the ITK inhibitor Cmpd-5 significantly diminished the proliferation of cancer cells enhanced by ITK expression; however, the ITK inhibitor had no adverse impact on the proliferation of mock control cells. These data suggest that ITK is a potentially useful novel molecular target for treating TSCC. Although there are no reports regarding clinical development of ITK inhibitors for treating epithelial cancers, ibrutinib, an inhibitor of the Tec kinase Bruton’s tyrosine kinase, has been used in practice for treating B-cell malignant lymphoma [35,36]. Clinical development of ITK inhibitors for treating autoimmune and allergic diseases is now also underway [37,38,39]. Inhibitors of Tec family kinases might prove applicable for treating TSCC in patients selected based on biomarkers indicating ITK overexpression. However, protein kinase inhibitors are known to affect other kinases [40]; thus, their specificity is important. When using ITK inhibitors clinically, it is necessary to extract more specific compounds. The ITK inhibitor used in this study was not optimized in clinical and preclinical studies. ADME (absorption, distribution, metabolism, and excretion) analyses required prior to administration to experimental animals in drug discovery research were not performed. Therefore, we could not carry out animal tests for the optimization of this compound. We plan to perform animal tests after ADME analyses of the related compounds.

Using an unbiased open reading frame-based screening approach to identify genetic modifiers of EGFR in EGFR-mutant non-small cell lung cancer (NSCLC), Sharifnia et al. recently showed that overexpression of ITK overcomes the suppression of cell proliferation with erlotinib, an EGFR inhibitor [41]. The resistance of oral cancers to anti-EGFR therapies could therefore be related to the overexpression of ITK, as indicated in the previous study of NSCLC.

ITK is phosphorylated by a lymphocyte-specific protein tyrosine kinase in T-cells after the peptide antigen binds to the T-cell receptor [2]. It has been reported that in CD8 T-cells, the amount of activated ITK differs depending on the affinity of the antigen [42], and in CD4 T-cells, the regulation of T-cell differentiation differs depending on the intensity of ITK activation [43]. It has also been reported that PTEN is involved as a negative regulator of ITK in T-cells [44], and the activity control mechanism of ITK in T-cells is very complex. Various modifications that occur within the tumor microenvironment have been shown to promote cancer cell growth [45]. It was also reported that annexin A6, which is not expressed in gastric cancer cells, is expressed in involved cancer-associated fibroblasts, and gastric cancer cells acquire treatment resistance [46]. In this experiment, ITK protein expression was confirmed in clinical samples but not in oral cancer cell lines. In clinical samples, ITK expression was confirmed due to the influence of the tumor microenvironment and stimulation, but it is possible that internal ITK expression could not be confirmed in oral cancer cell lines because the in vivo environment could not be reproduced. Furthermore, after confirming the interaction between GART and ITK with HEK 293 cells, a metabolomic analysis was performed in this study using oral cancer cells. Increases in GART and activity of the de novo purine biosynthesis pathway, which is a GART metabolic pathway, were confirmed in the ITK-expressing oral cancer cells but not in mock control cells. These results suggest an interaction between ITK and GART in oral cancer cells, but this interaction should be confirmed in the oral cancer cells in which ITK is originally expressed. These will be the subjects of future research.

A limitation of our study is that it remains unknown whether ITK-mediated phosphorylation of GART is directly related to enhanced de novo purine biosynthesis. Nevertheless, this is the first report demonstrating that ITK is associated with poor clinical outcome in TSCC and that ITK might be involved in enhancing the proliferation of cancer cells in the malignant phenotype via activation of de novo purine biosynthesis through phosphorylation of GART.

## 5. Conclusions

ITK is associated with poor clinical outcome in TSCC, and data suggest that ITK enhances the proliferation of cancer cells in the malignant phenotype via activation of de novo purine biosynthesis through phosphorylation of GART.

## Figures and Tables

**Table 1:** Association of ITK protein expression with clinicopathologic characteristics in stage I/II tongue squamous cell carcinoma.

	Number of Cases (%)	ITK-Negative	ITK-Positive	*p*-value ^a^
Total	86	73	13	
Age (years)				0.3655
<64	43 (50.0)	38	5	
≥64	43 (50.0)	35	8	
Sex				0.1715
Male	48 (55.8)	43	5	
Female	38 (44.2)	30	8	
Stage ^b^				0.9776
I	46 (53.5)	39	7	
II	40(46.5)	34	6	
Histologic differentiation				0.1760
Poor/Moderate	25 (29.1)	18	6	
Well	61 (70.9)	55	7	
Mode of invasion ^c^				**0.0000** *
1,2	58 (67.4)	56	2	
3,4	28 (32.6)	17	11	
Late metastasis of cervical lymph nodes				**0.0006** *
Negative	68 (79.1)	63	5	
Positive	18 (20.9)	10	8	
Lymphovascular invasion				**0.0101** *
Negative	75 (87.2)	67	8	
Positive	11 (12.8)	6	5	
Perineural invasion				
Negative	82 (95.3)	71	11	0.1067
Positive	4 (4.7)	2	2	

ITK, interleukin-2–inducible T-cell kinase. ^a^ Fisher’s exact test, * *p* < 0.05. Statistical significance associations are highlighted in bold. ^b^ According to the International Union Against Cancer (UICC) TNM Classification of Malignant Tumors, 8th edition. ^c^ Anneroth’s histological grading system.

**Table 2:** Hazard ratios for death with stage I/II tongue squamous cell carcinoma—Cox regression model.

Covariate	Univariate Analysis (*n* = 86)				Multivariate Analysis (*n* = 86)			
	HR	95% CI		*p*-Value	HR	95% CI		*p*-Value
Age (years)								
<64	Reference				Reference			
≥64	2.866	0.923	8.894	0.068	2.611	0.836	8.153	0.099
ITK protein expression								
Negative	Reference				Reference			
Positive	3.544	1.225	10.247	0.020 *	3.167	1.089	9.215	0.034 *
Sex								
Male	Reference							
Female	1.235	0.463	3.296	0.673				
Stage ^a^								
Stage I	Reference							
Stage II	1.519	0.567	4.068	0.405				
Histologic differentiation								
Well	Reference							
Poor and moderate	3.401	1.264	9.149	0.015 *				
Mode of invasion ^b^								
1, 2	Reference							
3, 4	5.112	1.768	14.783	0.003 *				
Late metastasis of cervical lymph nodes								
Negative	Reference							
Positive	4.497	1.684	12.010	0.003 *				
Lymphovascular invasion								
Negative	Reference							
Positive	3.497	1.210	10.110	0.021 *				
Perineural invasion								
Negative	Reference							
Positive	N.A.^c^							

HR, hazard ratio; CI, confidence interval; ITK, interleukin-2–inducible T-cell kinase. * *p* < 0.05. Statistical significance associations are highlighted in bold. ^a^According to the International Union Against Cancer (UICC) TNM Classification of Malignant Tumors, 8th edition ^b^ Anneroth’s histological grading system. ^c^ Could not be calculated.

## Data Availability

The data presented in this study are available on request from the corresponding author. The data are not publicly available due to privacy and ethical restrictions.

## Supplementary Materials

The following are available online at https://www.mdpi.com/article/10.3390/cancers13133333/s1, Figure S1: Chemical formula of the small compound used as an ITK inhibitor, Figure S2: Specificity of the anti-ITK antibody, Figure S3: Immunofluorescence cytochemistry of T-cells in TSCC tissue samples with anti-ITK (green) and anti-CD4 (red) antibodies, Figure S4: Immunofluorescence cytochemistry of SAS tumor cells of in vivo models stained with anti-ITK (green) and anti-Ki67 (red) antibodies, Figure S5: Tandem mass spectra representing phosphorylated residues in GART, Figure S6: Phosphotyrosine proteomic analysis of ITK-expressing cells and mock control cells treated with DMSO or ITK inhibitor, Table S1: Significantly higher levels of tyrosine-phosphorylated protein in ITK-expressing SAS cells than mock control cells, Table S2: Significantly higher levels of tyrosine-phosphorylated protein in ITK-expressing KOSC2 cells than mock control cells, Table S3: Significantly higher levels of 26 peptides common to ITK-expressing SAS and KOSC2 cells.
