# Supplementary material for: Possible Therapeutic Strategy Involving the Purine Synthesis Pathway Regulated by ITK in Tongue Squamous Cell Carcinoma
Source: Cancers (Basel). 2021 Jul 2;13(13):3333. doi: 10.3390/cancers13133333 (PMC8269312; doi:10.3390/cancers13133333)
Supplement: Supplementary file 1 [file cancers-13-03333-s001.zip › cancers-1284458_supp_for_XML.pdf]

Article

Possible Therapeutic Strategy Involving the Purine Synthesis Pathway Regulated by ITK in Tongue Squamous Cell Carcinoma

Kaoru Onidani, Nami Miura, Yuki Sugiura, Yuichi Abe, Yukio Watabe, Takanori Kakuya, Taisuke Mori, Seiichi Yoshimoto, Jun Adachi, Takao Kiyoi, Yasuaki Kabe, Makoto Suematsu, Takeshi Tomonaga, Takahiko Shibahara, and Kazufumi Honda

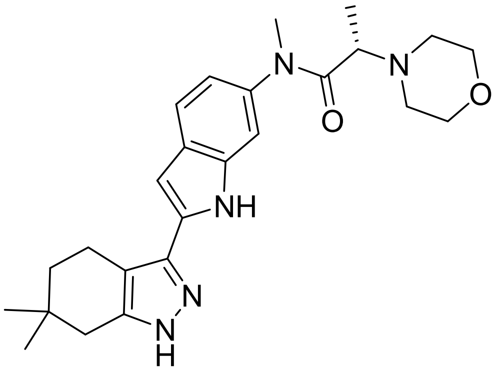

Chemical Formula: C25H33N5O2

Molecular Weight: 435.56

Figure S1. Chemical formula of the small compound used as an interleukin-2–inducible T-cell kinase (ITK) inhibitor. Chemical formula of the ITK inhibitor (Cmpd-5).

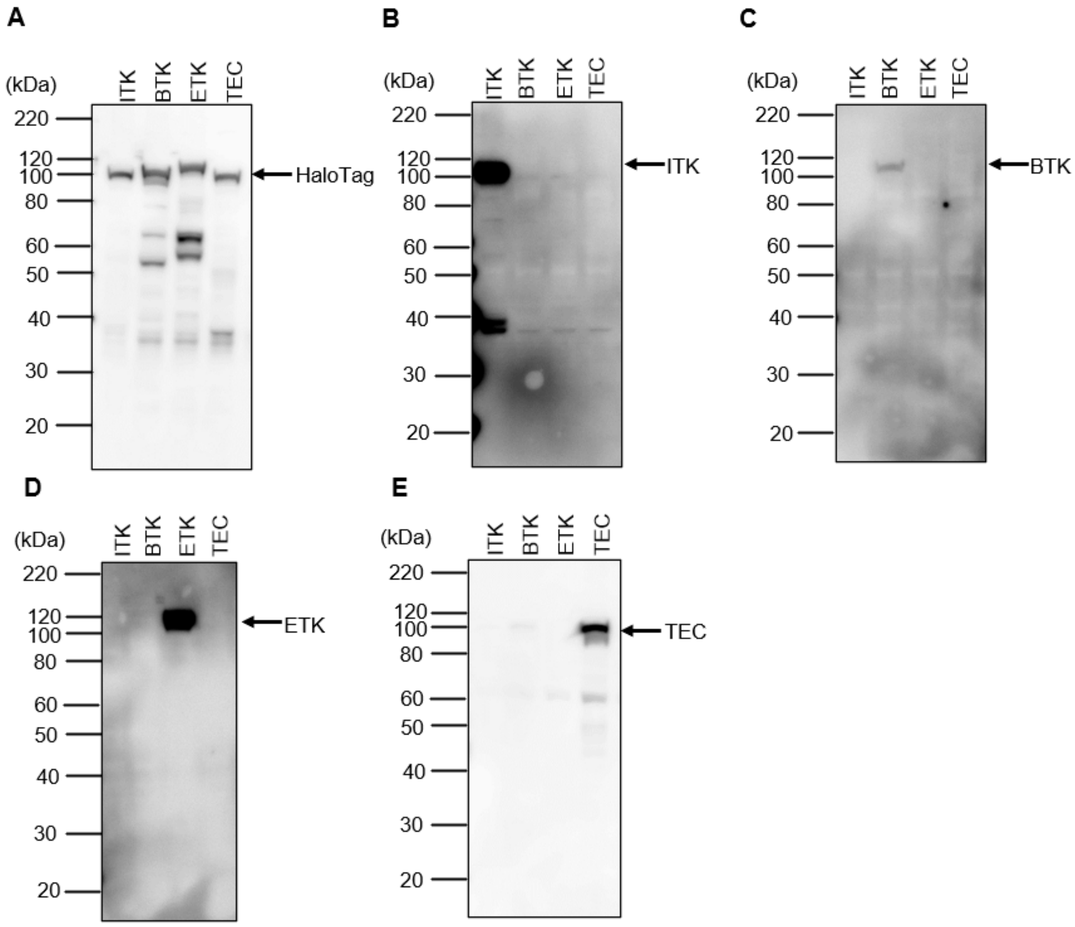

Figure S2. Specificity of the anti-interleukin-2–inducible T-cell kinase (ITK) antibody. (**A**–**E**) Western blot analysis of HaloTag (**A**), ITK (**B**), BTK, (**C**) ETK (**D**), and TEC (**E**) in HaloTag-fused ITK-, BTK-, ETK-, and Table 3. Phosphotyrosine proteomic analysis of ITK-expressing cells and mock control cells treated with DMSO or ITK inhibitor.

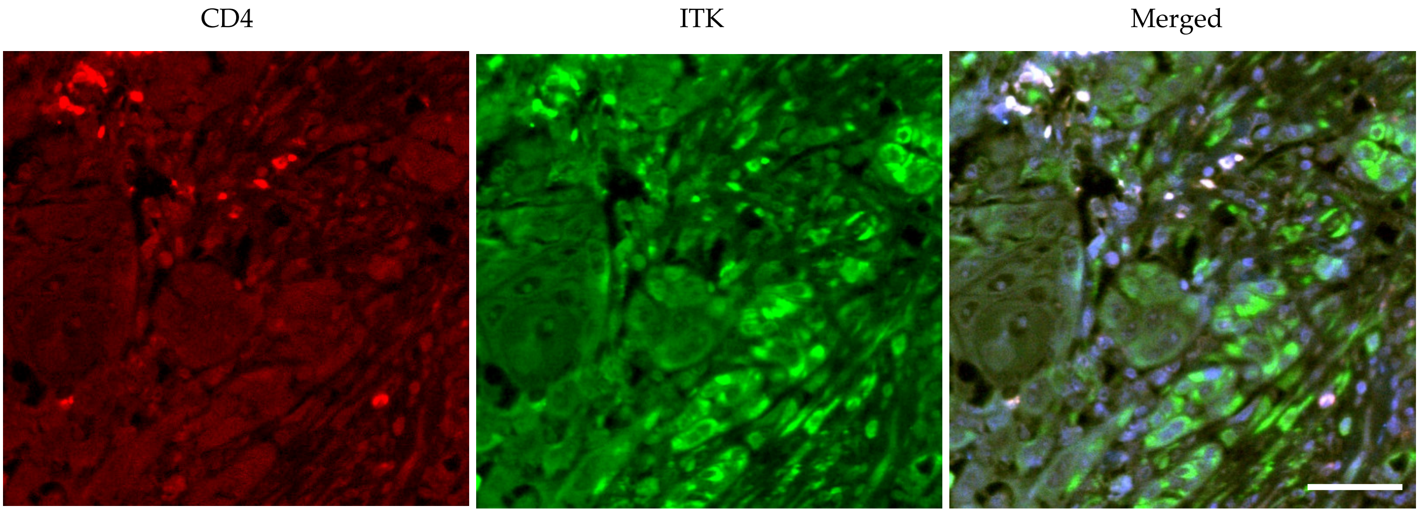

Figure S3. Immunofluorescence cytochemistry of T cells (arrows) in TSCC tissue samples stained with anti-interleukin-2–inducible T-cell kinase (ITK) (green) and anti-CD4 (red) antibodies.

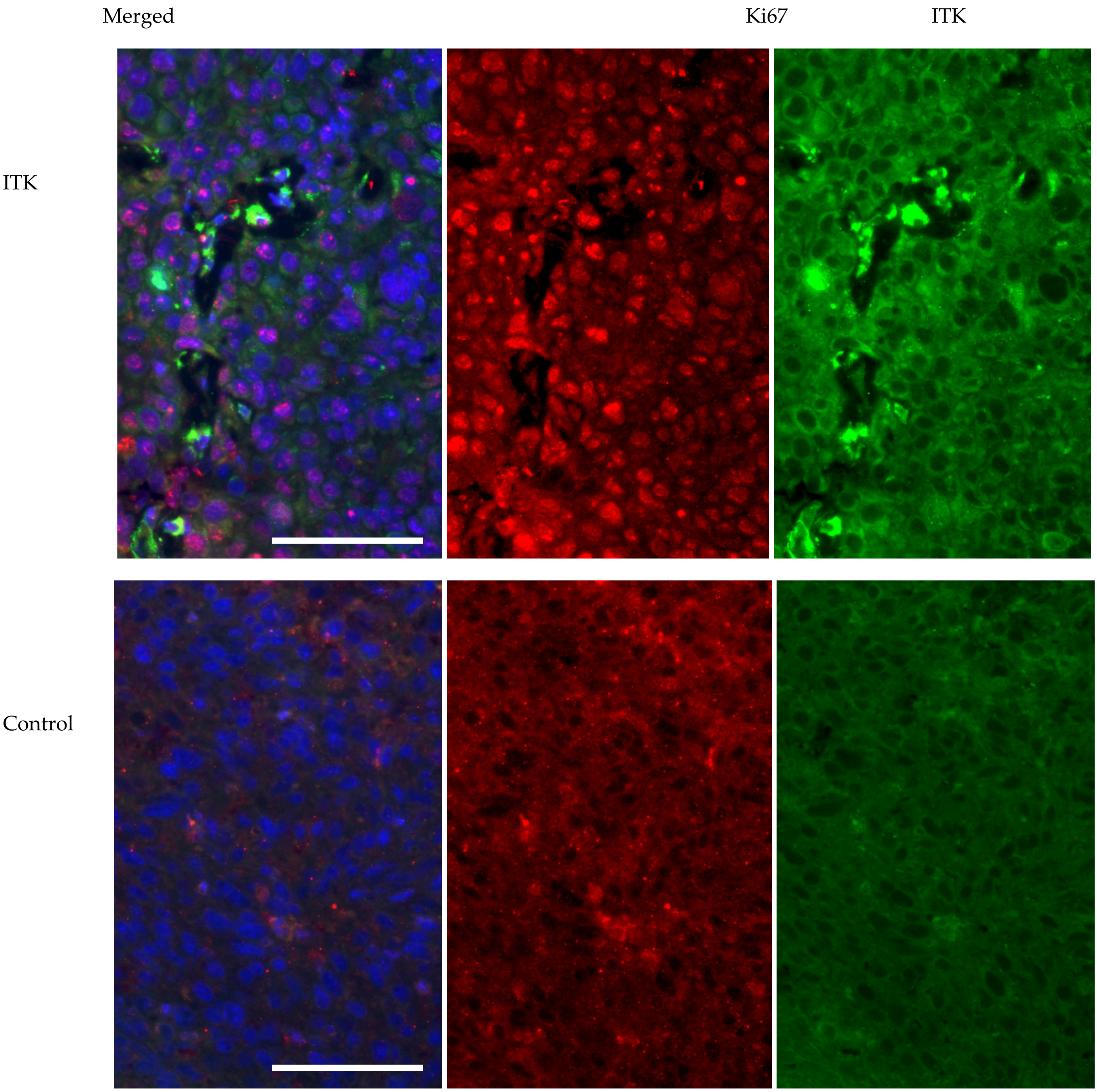

Figure S4. Immunofluorescence cytochemistry of SAS tumor cells of in vivo models stained with anti-interleukin-2–inducible T-cell kinase (ITK) (green) and anti-Ki67 (red) antibodies.

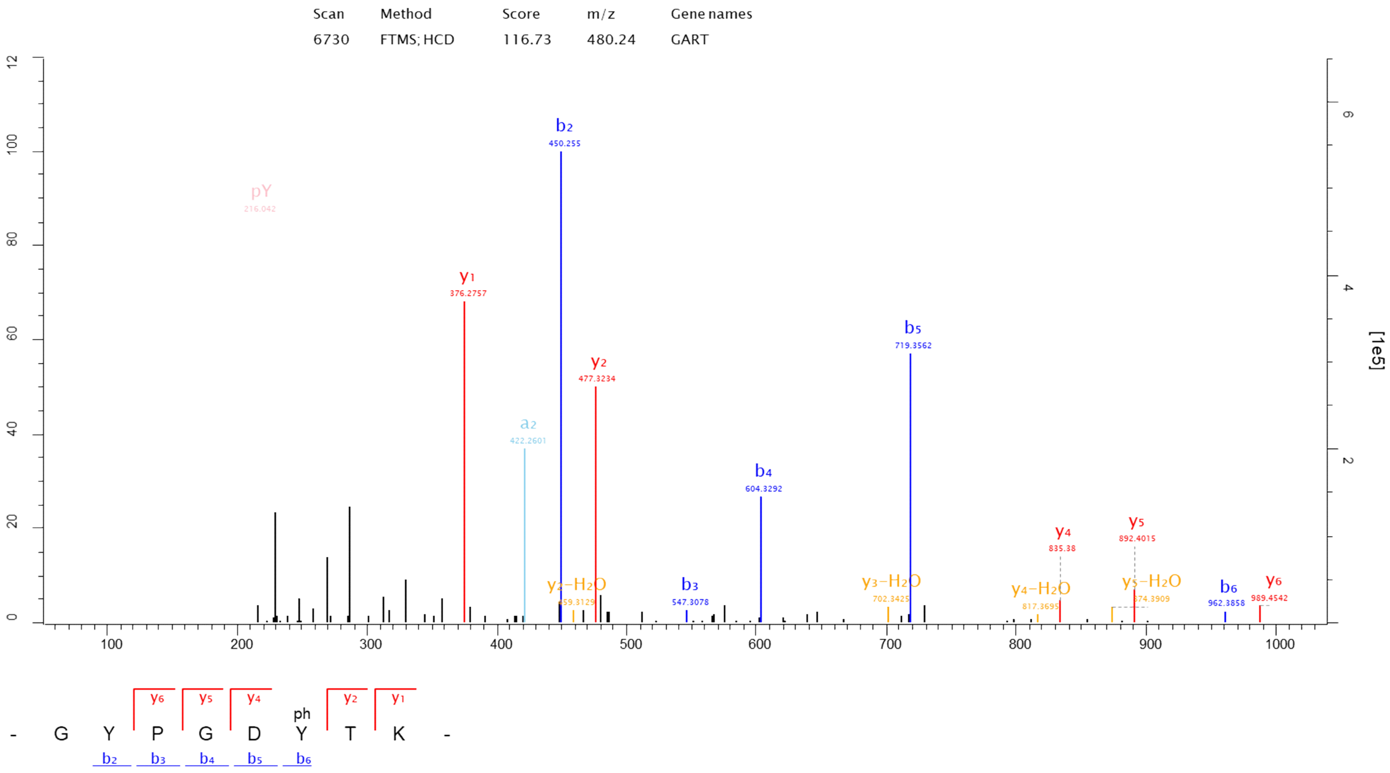

Figure S5. Tandem mass spectra representing phosphorylated residues in trifunctional purine biosynthetic protein adenosine-3 (GART). Spectra of phosphopeptides are color coded for matched b- and y-ions in blue and red, respectively. Tandem mass spectrum identifying pY348 of GART.

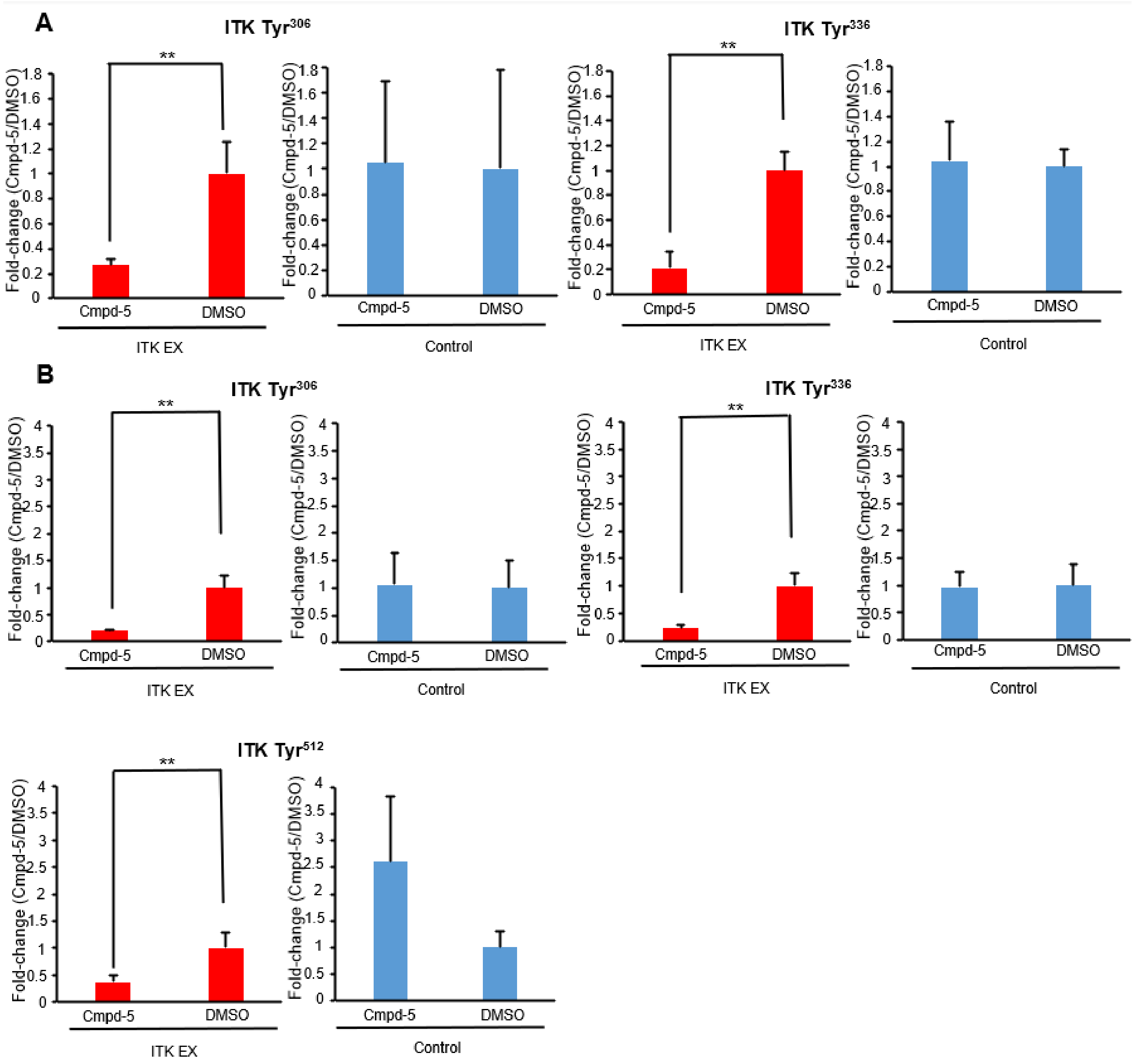

Figure S6. Phosphotyrosine proteomic analysis of interleukin-2–inducible T-cell kinase (ITK)-expressing cells and mock control cells treated with DMSO or ITK inhibitor. (A and B) ITK-expressing cells and mock control cells were treated with DMSO or ITK inhibitor (Cmpd-5) for 24 hours, and then phosphotyrosine residues on ITK peptides were analyzed (A: SAS cells; B: KOSC2 cells). ** $P < 0.05$ (Student's t-test).
