# Supplementary figures and images for: Possible Therapeutic Strategy Involving the Purine Synthesis Pathway Regulated by ITK in Tongue Squamous Cell Carcinoma
Source: Cancers (Basel). 2021 Jul 2;13(13):3333. doi: 10.3390/cancers13133333 (PMC8269312; doi:10.3390/cancers13133333)

Fig. 1K

ITK

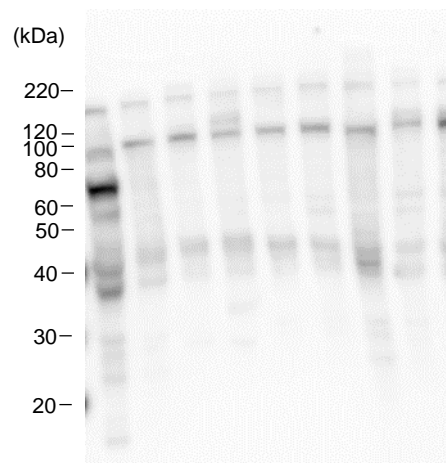

GART

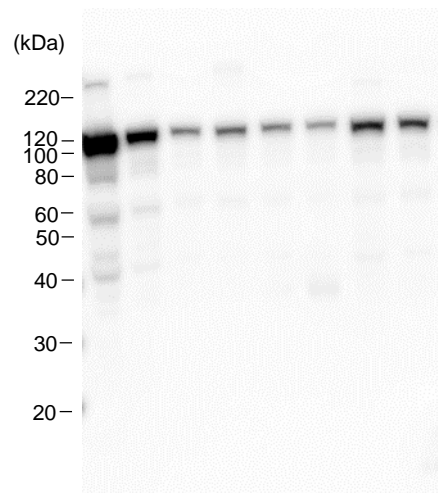

Bactin

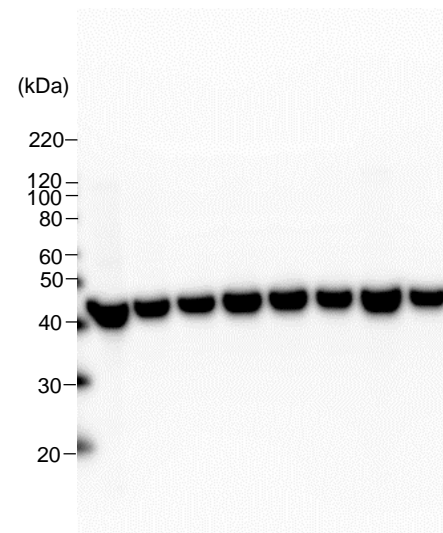

Fig. 1L

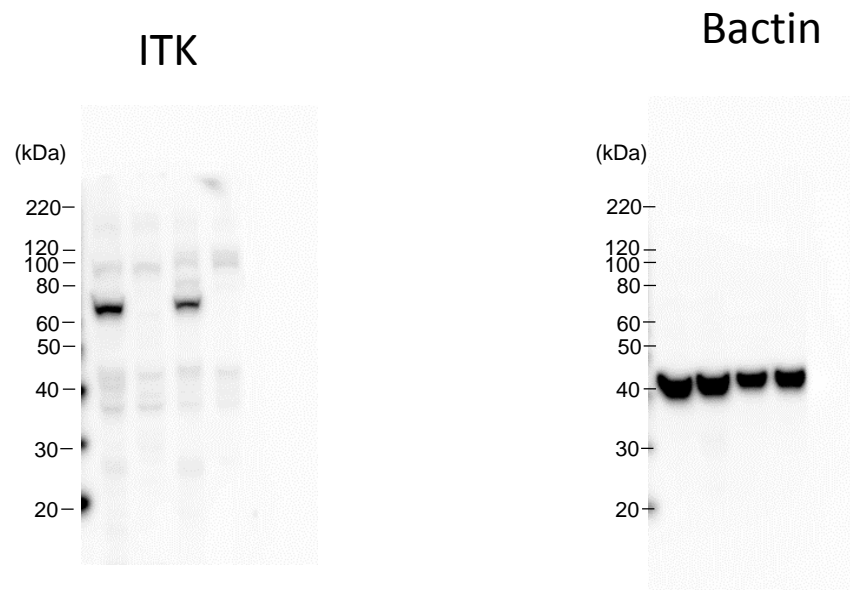

Fig. 2H

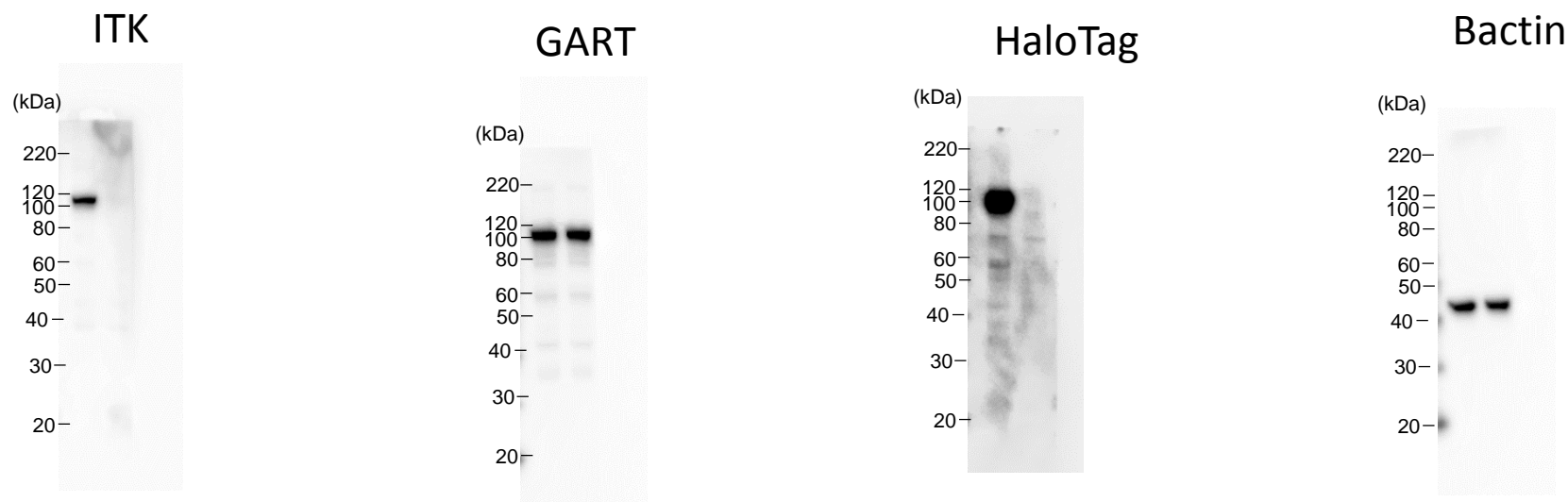

Fig. S2

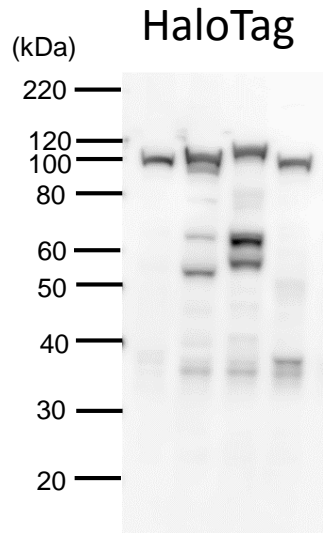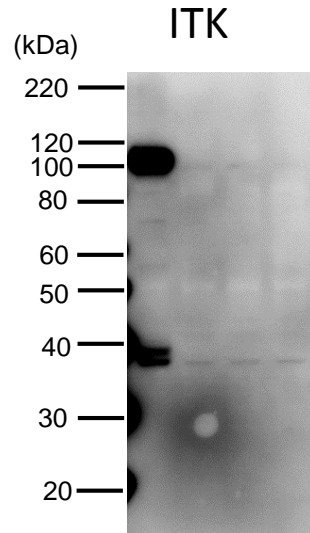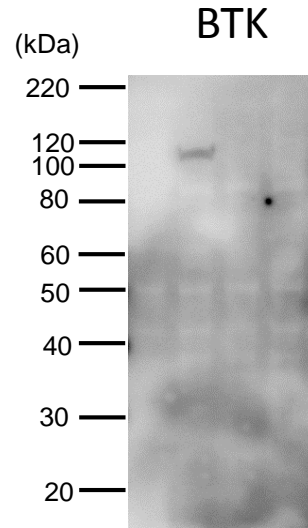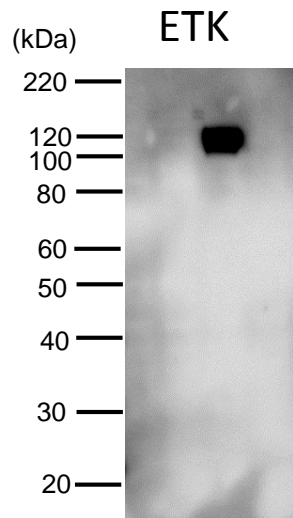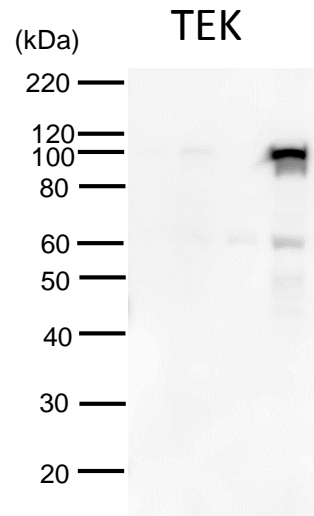

Supplement: Supplementary file 1 [file cancers-13-03333-s001.zip › Uncropped WB Figures.pdf]
